# Lipid pathways connecting maternal BMI with infant obesity risk

**DOI:** 10.1038/s41598-025-30081-7

**Published:** 2025-12-30

**Authors:** Alexandra D. George, Tingting Wang, Thy Duong, Yvette Schooneveldt, Sudip Paul, Gavriel Olshansky, Toby Mansell, Richard Saffery, Peter Vuillermin, Anne-Louise Ponsonby, David Burgner, Barwon Infant Study Investigator Group, Satvika Burugupalli, Peter J. Meikle

**Affiliations:** 1 Metabolomics Laboratory, Baker Heart and Diabetes Institute, Melbourne, VIC Australia; 2 Baker Department of Cardiometabolic Health, University of Melbourne, Parkville, VIC Australia; 3 Department of Cardiovascular Research, Translation and Implementation, La Trobe University, Bundoora, VIC Australia; 4 Murdoch Children’s Research Institute, Royal Children’s Hospital, Parkville, VIC Australia; 5 Department of Pediatrics, University of Melbourne, Parkville, VIC Australia; 6 School of Medicine, Deakin University, Melbourne, VIC Australia; 7 Child Health Research Unit, Barwon Health, Geelong, VIC Australia; 8 The Florey Institute of Neuroscience and Mental Health, Melbourne, VIC Australia; 9 Department of Diabetes, Central Clinical School, Monash University, Clayton, VIC Australia

**Keywords:** Lipid, DOHaD, Metabolic programming, Biomarkers, Diseases, Endocrinology, Health care, Medical research, Physiology, Risk factors

## Abstract

Maternal obesity is a key determinant of infant health, increasing early-life obesity risk. Lipids are mechanistically linked to obesity and may mediate intergenerational transfer, by influencing foetal or infant lipids. Using the Barwon Infant Study, we investigated associations between maternal pre-pregnancy body mass index (pp-BMI), lipidomic profiles of mothers, human milk, and infants, and early life growth. Ether lipids were of particular interest due to their abundance in human milk, association with breastfeeding, and roles in metabolism and inflammation. Linear regression analyses assessed relationships between maternal pp-BMI and lipid profiles across biospecimens, and infant BMI. A composite plasmalogen score, reflecting ether lipid metabolism, was developed due to its strong associations with pp-BMI and breastfeeding. Mediation analysis assessed if cord lipids mediated the effect of pp-BMI on birth weight. Maternal pp-BMI was significantly associated with maternal and cord lipids, and obesity risk indicators. Six cord blood lipids mediated up to 18% of the effect of pp-BMI on birth weight. Maternal plasmalogen score was negatively associated with pp-BMI and positively associated with human milk and infant plasmalogen scores from birth to four years of age. Infant plasmalogen score at six months was inversely associated with BMI z-score at four years of age. These findings suggest that ether lipids may be modifiable biomarkers of metabolic programming and intervention targets to reduce obesity risk in early life.

**Supplementary Information:**

The online version contains supplementary material available at 10.1038/s41598-025-30081-7.

## Introduction

The prevalence of overweight and obesity in children has reached alarming levels, affecting approximately one in four Australian children^1^. Contributing to this public health issue, maternal overweight and obesity are significant risk factors for infant obesity^2^. Maternal pre-conception obesity increases the odds of childhood obesity by up to 264%^3^ and maternal pre-pregnancy body mass index (pp-BMI) is associated with infant birth weight, BMI and overweight status^4,5^. Furthermore, high gestational weight gain is associated with increased infant BMI and obesity risk in adulthood^6^. These findings suggest that a healthy maternal metabolic status before and during pregnancy may contribute to favourable metabolic programming and reduced obesity risk for the infant, although the underlying biology remains poorly defined.

The role of lipids and lipid metabolism, known to be critical in metabolic health, has been largely overlooked in early life studies. Circulating lipids are known to have critical roles in health and disease and may be involved in health programming^7–9^. Adult obesity is associated with lipid dysregulation^8^, and during pregnancy, a time of high metabolic activity, many circulating lipids are elevated^7^. The circulating lipid profile in infancy changes across early life as metabolism develops, and previous studies have reported links between circulating lipids and growth in the first months and years of life^10,11^. Early life growth is also associated with breastfeeding, and the plasma lipid profiles of breastfed and formula-fed infants differ substantially^12^. Thus, lipid transfer from mother to infant may occur via the placenta in utero, or postnatally through human milk, providing two critical windows of exposure.

While this study considers the total circulating lipidome, we were particularly interested in ether lipids, a subclass of lipids characterised by an ether bond at the sn-1 position of the glycerol backbone. Ether lipids, including plasmalogens, are abundant in human milk, strongly associated with breastfeeding, and highly bioactive^12–14^. They play key roles in cellular membrane integrity, oxidative stress regulation, and immune development - functions that are critical during the early pre- and post-natal period^15^. Notably, ether lipids derived from human milk have been shown, in both mouse models and infant samples, to promote adipose tissue development and thermogenic capacity in early life^16^. Because ether lipids are modifiable through diet, they are compelling candidates for targeted interventions^17,18^. Despite their potential significance, ether lipids remain understudied in the context of early life metabolic programming and intergenerational obesity risk^19^.

Understanding how maternal lipids contribute to infant lipid profiles and obesity risk is critical. Circulating lipids are modifiable, and identifying key lipid pathways may reveal opportunities for targeted early interventions to reduce intergenerational obesity risk^7,12,20^. This study aimed to explore the role of maternal and infant lipids in the early-life transmission of obesity, using longitudinal data from the Barwon Infant Study^12,13,21^.

## Methods

### Study cohort and data

The Barwon Infant Study (BIS) cohort is a population-derived birth cohort from Victoria, Australia^21^. The cohort comprises 1074 mother-infant dyads and includes maternal and infant anthropometric measures. For this study all BIS mothers and infants with available lipidomics data and covariate data for each analysis were utilised, the subsets analysed were representative of the entire cohort (Table 1)^21^. Missing data were assumed to be missing at random, and each analysis was performed on all available cases. The distribution of maternal pp-BMI was as follows: 2.4% underweight (BMI < 18.5), 55.4% healthy (18.5 ≤ BMI < 25.0), 25.2% overweight (25.0 ≤ BMI < 30.0), and 17.0% obese (BMI ≥ 30.0). The mothers provided written informed consent at recruitment and study ethics was approved by the Barwon Health Human Research Ethics Committee (HREC 10/24). All experiments were performed in accordance with the relevant guidelines and regulations. Comprehensive lipidomics profiling using liquid chromatography-mass spectrometry has previously been performed on infant plasma, cord blood, maternal serum, and maternal milk in the BIS. Briefly, 733 lipid species were measured in maternal samples at 28 weeks’ gestation, cord serum, and infant plasma at 6, 12, and 48 months, and > 900 lipid species in maternal milk samples at 1 and 6 months^12,13^.

**Table 1:** The basic characteristics of BIS cohort variables utilised in this study.

Variable	Variation^1^	Data completeness (%)
Maternal characteristics		
Pre-pregnancy BMI (kg/m^2^)	25.7 (5.6)	63
Age (years)	31.9 (4.5)	63
University education (yes)	460 (53.6%)	80
Infant characteristics		
Sex (female)	495 (48.5%)	95
Gestational age (days)	276.5 (10.2)	95
Birth weight (kg)	3.6 (0.5)	92
BMI (kg/m^2^)		
At 6 months	17.2 (1.7)	79
At 12 months	17.7 (1.7)	77
At 48 months	15.6 (1.4)	62
Birth mode		
Unassisted vaginal birth^2^	510 (50.1%)	95
Unscheduled caesarean section	144 (14.1%)	
Forceps vaginal birth	86 (8.4%)	
Scheduled caesarean section	160 (15.7%)	
Vacuum vaginal birth	119 (11.7%)	
Breastfeeding (any) duration (weeks)	28.65 (21.1)	95
Breastfeeding status (yes)		
At 6 months	549 (60.3%)	85
At 12 months	286 (31.4%)	85

^1^Values are presented as mean (standard deviation) for continuous variables or number (percentage) for categorical variables; BMI: body mass index, ^2^Reference category.

### Statistical analyses

**Fig. 1:**
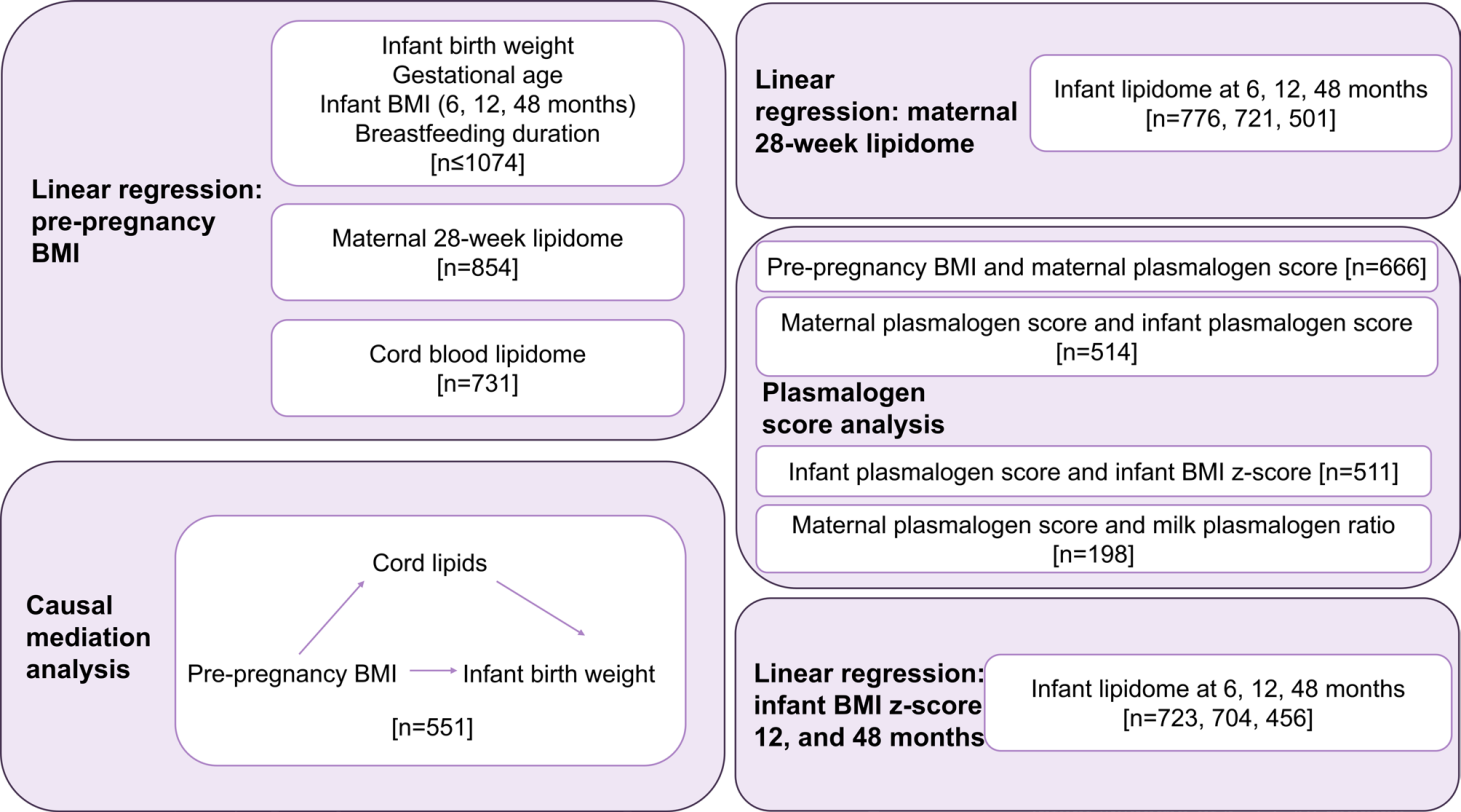
Overview of statistical analyses to interrogate the lipid link between maternal obesity and infant obesity risk in the Barwon Infant Study cohort.

An overview of the analyses in this study is presented in Fig. 1.

Variables of interest and covariates utilised throughout the analyses were maternal pp-BMI (kg/m^2^), maternal age (years), birth mode (categorical), infant gestational age (weeks), infant birth weight (kg), infant BMI (kg/m^2^), infant BMI z-scores (calculated from WHO child growth standards using infant weight, length, age, and sex), maternal clinical non-fasted lipids (high-density lipoprotein, HDL, cholesterol, triglycerides at 28 weeks), breastfeeding duration (weeks ≤ 52, defined as any breastfeeding, self-reported), breastfeeding at 6 months and 12 months (binary yes or no at each time point), and maternal education (dichotomised as completed any university level certificate or less than university level education). Sample size differs for each analysis, due to incomplete data and/or samples.

Lipidomics measures were log transformed and scaled to unit variance prior to analysis. Associations between pp-BMI and infant birth weight, gestational age, infant BMI at 6, 12, and 48 months, and breastfeeding duration were assessed using univariate linear regression with no covariates. Associations between pp-BMI and maternal lipidome were assessed using multivariable linear regression, adjusted for maternal age, education, and clinical lipids (*n* = 673). Clinical lipids (total cholesterol, HDL-cholesterol, triglycerides) were included as covariates, consistent with standard practice in adult lipidomics studies, to assess associations of individual maternal lipid species independent of underlying lipoprotein concentrations. The fold change for maternal lipids associated with pp-BMI were included in the supplementary material of a previous publication^12^. Associations between pp-BMI and the cord lipidome were assessed using multivariable linear regression, adjusted for maternal age, infant birth weight, delivery mode, infant sex, and gestational age (*n* = 668). Associations between paired maternal and infant lipids at each time point were assessed using multivariable linear regression, adjusted for both maternal BMI and infant BMI z-score, infant sex, birth weight, and breastfeeding (*n* = 501–776). Associations between infant BMI z-score and infant lipidomes at 6, 12, and 48 months were assessed using multivariable linear regression, adjusted for infant sex, and breastfeeding (*n* = 456–723). Covariate selection in each adjusted model was complicated, and informed by previous BIS work and population lipidomics, focusing on established confounders^8,12,18^. Each multivariable regression analysed lipid species individually, and all p-values were corrected for multiple comparisons by the method of Benjamini-Hochberg (BH)^22^.

Causal mediation analysis was performed using “mediation” R package, to investigate the mediating effect of cord lipids on the relationship between maternal pp-BMI and infant birth weight, a well-established early indicator of later obesity risk (adjusted for infant sex, delivery mode, gestational age, and maternal age)^23^. Total effects were estimated from the linear regression analysis. There was limited evidence to support performing other mediation analyses in this study (i.e. mediation was only tested when the exposure was significantly associated with both the mediator and the outcome). For each lipid, two models were constructed: (1) a mediator model with the lipid as the dependent variable and pp-BMI as the independent variable, and (2) an outcome model with birth weight as the dependent variable and both pp-BMI and the lipid as independent variables. The “mediate” function was used to quantify the indirect (mediated) effect, direct effect, and total effect of pp-BMI on birth weight. Mediation models were fitted using non-parametric bootstrapping with 10,000 simulations to estimate 95% confidence intervals for the proportion of the effect mediated by each lipid (*n* = 551). Cord lipids that did not meet the mediation requirements of being associated with maternal pp-BMI, or had negative mediation results, were considered invalid and excluded.

To provide a composite lipid measure and reduce the difficulty in interpreting individual lipid species, we developed a plasmalogen score. This score was derived via principal component analysis (PCA) on the mean-centred and scaled phosphatidylethanolamine plasmalogen (PE(P)) and phosphatidylethanolamine (PE) species, from each expressed as a proportion of total PE(P) and PE. The first principal component (PC1) was used as the plasmalogen score, separately calculated for maternal serum at 28 weeks’ gestation and infant plasma at 48 months. This plasmalogen score was selected because it provides a biologically interpretable value, which has been previously validated in adult cohorts in relation to cardiometabolic outcomes, to ensure the approach was biologically meaningful. This plasmalogen score is strongly associated with BMI in adults^18^. We hypothesised that a plasmalogen score might also represent a meaningful biological signature of early lipid programming. For infant plasma at 6 and 12 months, the 48-month infant PCA model was applied to calculate plasmalogen scores, avoiding the confounding due to breastfeeding at 6- and 12-month timepoints. For human milk samples, a plasmalogen ratio was calculated (total PE(P)/total PE) instead of a PCA-based score due to compositional differences in lipid species between milk and plasma. Linear regression models were used to examine the following associations: (1) maternal pp-BMI and plasmalogen score (adjusted for maternal age, *n* = 666); (2) maternal plasmalogen score and infant plasmalogen score (adjusted for maternal age, birth weight or BMI z-score, breastfeeding, and maternal pp-BMI, *n* = 514); (3) infant plasmalogen score and infant BMI (adjusted for maternal age, breastfeeding, birth weight, and maternal pp-BMI, *n* = 511); (4) maternal plasmalogen score and human milk plasmalogen ratio (adjusted for maternal age and pp-BMI, *n* = 198). Analyses were performed in RStudio (version 2024.04.1). For each model, statistical significance was defined as *p* < 0.05, either unadjusted or after BH correction.

## Results

### Factors associated with maternal pre-pregnancy BMI

We examined associations between maternal pp-BMI and obesity related outcomes in the Barwon Infant Study (BIS) cohort; infant birth weight and age, BMI, and duration of breastfeeding (Table 2).

**Table 2:** Associations of maternal pre-pregnancy body mass index (kg/m^2^) with infant growth and breastfeeding.

Outcome	Beta coefficient [95% CI]	*p*-value^1^
Birth weight (kg)	0.013 [0.007, 0.020]	**9.12 × 10** ^**− 5**^
Gestational age (days)	0.0001 [-0.0.043, 0.043]	9.87 × 10^− 1^
BMI at 6 months (kg/m^2^)	0.052 [0.028, 0.076]	**2.99 × 10** ^**− 5**^
BMI at 12 months (kg/m^2^)	0.056 [0.031, 0.081]	**9.48 × 10** ^**− 6**^
BMI at 48 months (kg/m^2^)	0.051 [0.028, 0.073]	**9.53 × 10** ^**− 6**^
Breastfeeding duration (weeks)	-0.937 [-1.193, -0.680]	**1.95 × 10** ^**− 12**^

^1^significant p-values (*p* < 0.05) are in bold; BMI: body mass index. No model adjustments were made.

We used maternal pp-BMI as a proxy for maternal obesity, with higher pp-BMI representing increased risk of infant obesity. Infant birthweight and BMI at 6, 12, and 48 months were used as indicators of early-life obesity risk, with higher values interpreted as proxies for greater risk. Breastfeeding duration, and associations with breastfeeding, were considered indicators of protection against obesity. These variables were used to investigate how lipid profiles may contribute to the intergenerational transfer of obesity risk or resilience.

### Maternal pre-pregnancy BMI is associated with gestational lipids and cord blood lipids

**Fig. 2:**
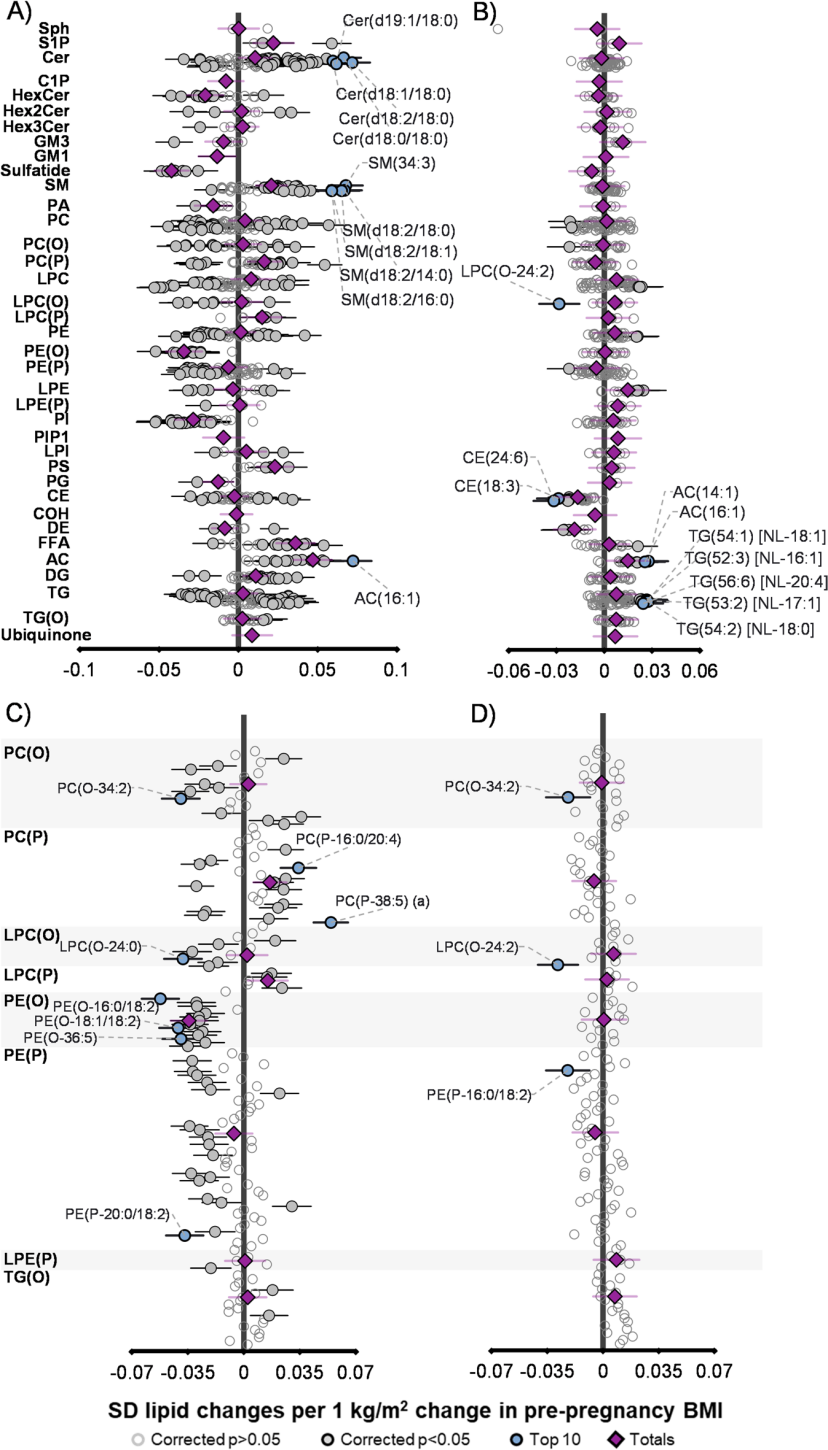
Associations between maternal pre-pregnancy BMI and lipid species in gestational and cord blood. Forest plots display regression coefficients for associations of maternal pre-pregnancy BMI with (**A**) maternal gestational lipids, (**B**) cord blood lipids, (**C**) maternal ether lipids, and (**D**) cord blood ether lipids. For maternal lipids, analysis was adjusted for maternal age, education, and clinical lipids (*n* = 673). For cord blood lipids, analysis was adjusted for infant birth weight, delivery mode, sex, and gestational age (*n* = 668). Each circle represents an individual lipid species, open circles represent *p* > 0.05, white closed grey circles represent corrected *p* < 0.05, the top 10 most significantly associated lipid species are shown in blue and labelled. Purple diamonds represent lipid class totals. All p-values were corrected for multiple comparisons (BH correction). Horizontal bars indicate 95% confidence intervals for lipid class totals and significant lipid species. Abbreviations for lipids are: acylcarnitine (AC), alkenylphosphatidylcholine (PC(P)), alkenylphosphatidylethanolamine (PE(P)), alkylphosphatidylcholine (PC(O)), alkylphosphatidylethanolamine (PE(O)), alkyldiacylglycerol (TG(O)), ceramide (Cer), ceramide-1-phosphate (C1P), cholesterol ester (CE), dehydrocholesterol ester (DE), diacylglycerol (DG), dihexosylceramide (Hex2Cer), cholesterol (COH), free fatty acid (FFA), GM_1_ ganglioside (GM1), ganglioside GM_3_ (GM3), lysophosphatidylcholine (LPC), lysoalkylphosphatidylcholine (LPC(O)), lysoalkenylphosphatidylcholine (LPC(P)), lysophosphatidylethanolamine (LPE), lysoalkenylphosphatidylethanolamine (LPE(P)), lysophosphatidylinositol (LPI), monohexosylceramide (HexCer), phosphatidic acid (PA), phosphatidylcholine (PC), phosphatidylethanolamine (PE), phosphatidylglycerol (PG), phosphatidylinositol (PI), phosphatidylinositol-1-phosphate (PIP1), phosphatidylserine (PS), sphingomyelin (SM), sphingosine (Sph), sphingosine-1-phosphate (S1P), triacylglycerol (TG), and trihexosylceramide (Hex3Cer).

As BMI and the plasma lipidome are linked in non-pregnant populations^8^, we confirmed that maternal pp-BMI was associated with the plasma lipidome during gestation^12^ (Supp Table 1). The maternal 28-week gestational lipidome showed significant associations with pp-BMI after correcting for multiple comparisons (50.1%, 367/733 lipid species, Fig. 2A). Approximately half (182/367) of the lipids were negatively associated with maternal pp-BMI, including several ether lipids from classes PE(P) and PE(O) (Fig. 2C). The remaining (185/367) lipids were positively associated with maternal pp-BMI, including many species from the ceramide, sphingomyelin, acylcarnitine, and glycerolipid classes. We also assessed if maternal pp-BMI was linked to cord lipids (Supp Table 2). The cord blood lipidome contained 41 lipids significantly associated with maternal pp-BMI after BH correction (Fig. 2B). These included 41% (17/41) negatively associated (from CE, LPC(O), DE, PC(O), PE(P), Fig. 2D) and 59% (24/41) positively associated, predominantly from TG, AC, and FFA classes. In contrast, maternal pp-BMI was significantly associated with only one infant lipid (TG(56:8) at 6 months of age).

### Cord lipids partially mediate the effect of maternal pre-pregnancy BMI on infant birth weight

**Fig. 3:**
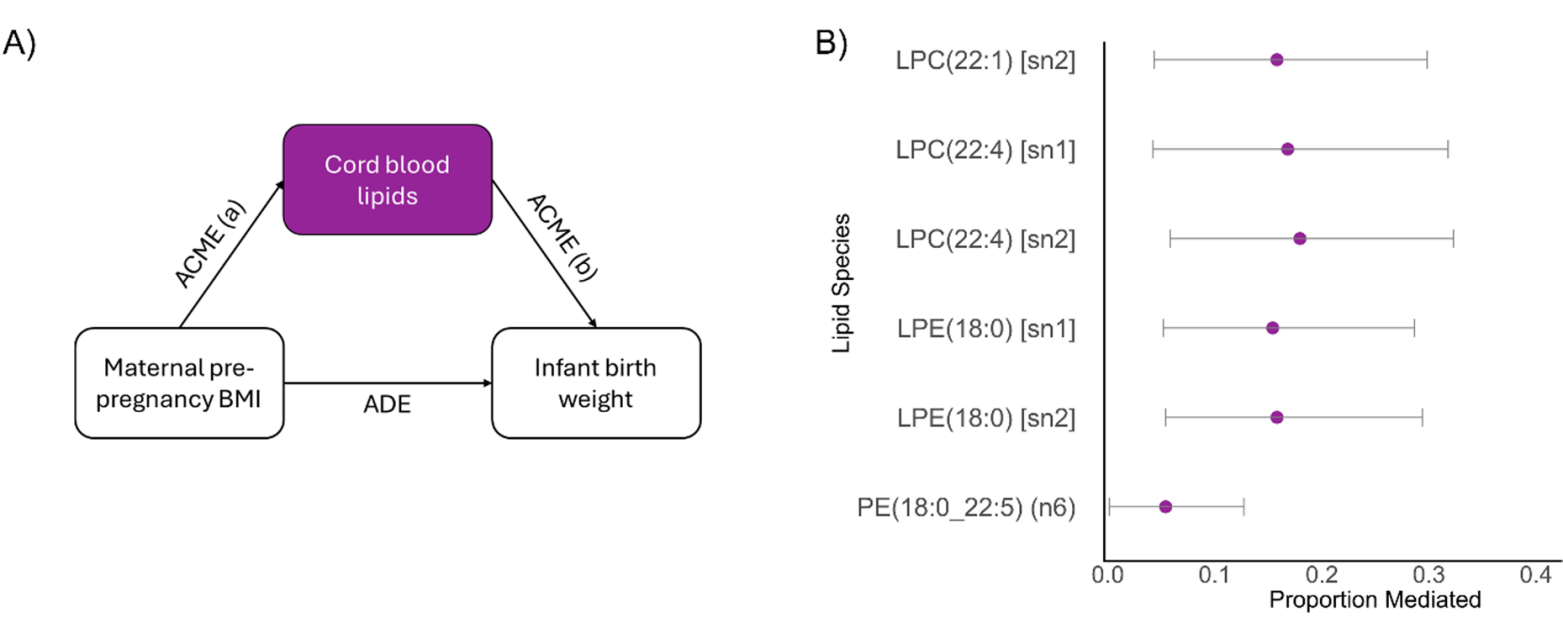
Cord lipids mediate some of the association between maternal pre-pregnancy BMI and infant birth weight. (**A**) Proportion mediation was estimated in causal mediation models, where maternal pp-BMI was the exposure, infant birth weight was the outcome, and cord lipids were the mediator (adjusted for maternal age, infant gestational age, infant sex, and delivery mode). Average causal mediation effect (ACME) = product of the associations between the exposure and mediator, and the mediator and outcome; Total effect = average direct effect (ADE) + ACME; Proportion mediated = ACME/ Total effect. (B)** C**ord lipids that significantly mediate the association of maternal pp-BMI on birth weight, proportion mediated is plotted with 95% confidence intervals.

Having established associations between maternal pp-BMI and infant birth weight, and maternal pp-BMI and cord lipids, we performed mediation analysis to investigate the extent to which cord lipids mediate the effect of pp-BMI on infant birth weight (Supp Table 3). There were 6 lipids (Fig. 3) that mediated between 5.4 and 18.0% of the effect of maternal pp-BMI on birth weight, after BH correction.

### Associations between lipids, plasmalogen scores, and obesity-related outcomes

To explore potential protective lipid mechanisms linked to maternal pp-BMI, breastfeeding, and infant growth, we looked at lipids, focusing on ether lipids, and calculated a composite plasmalogen score. We aimed to identify how lipids may act across the maternal-infant axis to influence obesity risk protectively. We first assessed associations between infant lipid species and BMI z-score at 6, 12, and 48 months. At 6 months of age, 20 lipid species were significantly associated with infant BMI z-score (*p* < 0.05). In contrast, only one lipid was significantly associated with BMI at 12 and 48 months (Supplementary Tables 4–6, Supplementary Figs. 1–3). Then we checked the association between maternal and infant lipids at all timepoints, which showed frequent significantly positive association of ether lipid species, particularly plasmalogens (Supplementary Tables 7–9).

Plasmalogen scores were then calculated in maternal serum and infant plasma, cord blood, and the plasmalogen ratio in maternal milk. Linear regression was used to examine associations between maternal pp-BMI and maternal plasmalogen score, maternal plasmalogen score and milk plasmalogen ratio, maternal and infant plasmalogen scores, and infant plasmalogen score and infant BMI, including appropriate covariates as in the previous models (Table 3, Supp Table 10). Maternal plasmalogen score at 28 weeks’ gestation was positively associated with plasmalogen score at birth, 6, 12, 48 months of age, and the human milk plasmalogen ratio. Longitudinally, infant 6-month plasmalogen score was negatively associated with BMI z-score at 48 months. Maternal 28 weeks’ gestation weight and weight gain were also explored as measures for maternal obesity; however, these were not included in the final models (Supp 11).

**Table 3:** The plasmalogen score link between maternal BMI and infant BMI.

Association	Effect estimate (β)	95%CI	*p*-value^1^	*R*-squared
Pre-pregnancy BMI with maternal plasmalogen score	-0.116	-0.196, -0.035	**4.94 × 10** ^**− 3**^	0.011
Maternal plasmalogen score with cord blood plasmalogen score	0.789	0.529, 1.049	**4.47 × 10** ^**− 9**^	0.047
Maternal plasmalogen score and milk plasmalogen ratio	0.765	0.044, 1.487	**3.77 × 10** ^**− 2**^	0.020
Maternal plasmalogen score with infant 6-month plasmalogen score	0.218	0.008, 0.427	**4.22 × 10** ^**− 2**^	0.408
Maternal plasmalogen score with Infant 12-month plasmalogen score	0.412	0.226, 0.598	**1.69 × 10** ^**− 5**^	0.251
Maternal plasmalogen score with child 48-month plasmalogen score	0.201	0.096, 0.309	**3.77 × 10-** ^**2**^	0.031
Infant 6-month plasmalogen score and 48-month BMI z-score	-0.237	-0.443, -0.030	**2.50 × 10** ^**− 2**^	0.419

^1^significant p-values (*p* < 0.05) are in bold.

## Discussion

Maternal obesity is a major risk factor for infant obesity, yet the biological mechanisms remain unclear^24^. This study reveals that lipid metabolism, including ether lipids, may serve as a key protective factor capable of modifying the intergenerational transmission of obesity risk.

### Maternal pre-pregnancy BMI and protective lipid signatures

Maternal pp-BMI, which is a key determinant of infant obesity risk^2^, was associated with a distinct lipid signature during pregnancy (28 weeks’ gestation, Fig. 2A). Higher maternal BMI at conception has consistently been linked with increased risk of macrosomia and childhood obesity and was therefore used as a proxy for obesity^2,3,5,24^. Consistent with this, we observed a positive association between maternal pp-BMI and infant birth weight, which is a known risk factor for later obesity^25^. While maternal dysregulation of free fatty acid (FFA) metabolism has been commonly implicated in transfer of obesity risk to the infant^26^, we observed that many other lipid classes were also affected. This included several increased ceramides and sphingolipids, and decreases to hexosylceramides, sulfatides, and many ether lipids and the maternal plasmalogen score. Similar dysregulation is also observed in non-pregnant adults, including the negative relationship between BMI, type 2 diabetes, and cardiovascular disease with plasmalogen score^9,18^. The plasmalogen score allows the assessment of relative PE(P) and PE levels, is modifiable, and has been previously validated as a metabolic marker for adult cardiometabolic health^18^. The maternal lipid profile with lower pp-BMI, marked with higher ether lipid and plasmalogen levels, may reflect a protective metabolic signature for pregnancy. This signature may contribute to healthy offspring growth and development (for example a less adverse birth weight) but is diminished with a higher maternal pp-BMI. Overall, these findings suggest that maternal lipid profiles characteristic of obesity have the potential to be passed on to the newborn, via the umbilical cord in utero, and via human milk postnatally, ultimately setting-up metabolism and future obesity risk.

### Cord blood lipids as early mediators of obesity risk and protection

Maternal pp-BMI can alter the cord blood lipids, which represent infant circulating lipids at birth (Fig. 2B). While transfer of polyunsaturated fatty acids (PUFA) from mother to foetus is tightly regulated^27,28^, our findings show that maternal metabolic status is still reflected in the infant birth lipidome. There were 6 cord lipids, comprising LPCs, LPEs, and one PE species, that each significantly mediated up to 18.0% of the relationship between maternal pp-BMI and infant birth weight (Fig. 3). Many lysophospholipids have previously been linked to adult obesity and infant growth^29^. This suggests that higher pp-BMI is associated with a shift away from a more protective cord lipid profile, reflective of maternal circulation, and towards a lipid state linked with adversely high infant birth weight^25^. It is possible that other lipids that are dysregulated by maternal obesity may also exert effects in utero, for example, in a prior BIS analysis, several ether lipids, including PC(P) and TG(O) species, were negatively associated with birth weight^12^, and studies in placental tissue have shown impaired transfer of ether lipids in pregnancies complicated by obesity^30^. Further exploring lipids, particularly modifiable lipids, in this context will be important to elucidate pathways through which maternal metabolic status programs offspring health.

### Enhancing maternal plasmalogen score to reduce infant obesity risk

Our results highlight that increasing maternal ether lipid levels and plasmalogen score may offer a novel intervention to promote healthier lipid profiles and influence infant metabolic outcomes. This could be achieved through maternal dietary modification and/or reduction of pp-BMI to a healthy range^31,32^. Reducing BMI, which is widely recommended for women with overweight or obesity during pregnancy planning, is likely to improve lipid profiles and infant health outcomes^33,34^. Additionally, our findings suggest that lipid supplementation of women prior to/during pregnancy, especially in women with high BMI, to normalise lipid profiles could be trialled to reduce infant obesity risk, as maternal and infant plasmalogen scores were positively associated independent of maternal pp-BMI. Human studies have shown that plasmalogens are modifiable through alkylglycerol precursors or plasmalogens^35–37^. We have previously shown in the BIS that the majority of cord blood lipids are significantly positively associated with maternal gestational lipids, implying that modification of the maternal lipidome during pregnancy may modify cord blood lipids^12^. Maternal dietary supplementation with plasmalogen precursors, such as alkylglycerols, may promote a gestational lipid profile associated with lower infant obesity risk by enhancing protective ether lipid signals in both maternal and cord blood, supporting protective lipid transfer to the infant.

### The impact of maternal obesity on developing infant lipid metabolism

Postnatally, we observed significant correlations between maternal and infant plasmalogen scores, at 6, 12, and 48 months of age, suggesting transmission of ether lipid profiles across maternal and infant contexts. This is a novel finding and, to our knowledge, one of the first demonstrations of maternal-infant lipid tracking across timepoints with a composite lipid (plasmalogen) score. The strong associations between maternal and infant plasmalogen scores up to four years of age, even after adjusting for breastfeeding, suggests that although human milk is a major source of ether lipids in infancy, maternal lipid metabolism may exert a longer-term influence on the infant lipid profile beyond the breastfeeding window. As the child ages, shared dietary exposures between mother and infant are also likely to contribute to the observed associations; however, dietary sources of plasmalogens in humans are limited, and we did not have detailed dietary data to disentangle these effects.

### Early life lipid profiling to understand obesity risk

Understanding early life lipid metabolism is essential for translating these findings into strategies that support healthy development and reduce obesity risk. While higher BMI in infancy is associated with increased risk of obesity later in life, BMI is a crude measure of metabolic health during infancy^38–40^. Our data reveal that associations between lipids and BMI at 6 and 12 months were weak and inconsistent, but by 4 years of age, the lipidomic profile began to reflect adult-like patterns. This suggests a developmental shift in lipid metabolism and highlights the importance of long-term follow-up in birth cohorts to fully capture when and how lipid signatures predict obesity risk. Among the lipid classes, ether lipids, particularly plasmalogens, are emerging as key candidates in early life metabolic priming^12,13,16,41^. Plasmalogens, higher in mothers with lower pp-BMI, may represent a biological link between maternal metabolic status, infant lipid programming, and obesity risk. Mechanistic studies support this role, for example in mice dietary alkylglycerols (plasmalogen precursors) enhance mitochondrial activity, promote beige adipose tissue, and activate lipid-regulating pathways^16^. In humans, early ether lipid intake has been linked to both increased weight gain in infancy and reduced fat mass, underscoring their dynamic influence on growth^42^. Our data suggest that the plasmalogen score could serve as a biologically meaningful lipid marker of protective potential in the context of intergenerational obesity risk. The score reflects maternal metabolic status, is associated with milk composition, persists across infancy, and begins to show metabolic relevance by 4 years of age. Although a number of individual lipid species were also significantly associated with maternal BMI and infant outcomes, we chose to prioritise plasmalogens because they represent a biologically plausible pathway with established links to obesity and metabolic health, and importantly, they are modifiable in early life. Infant plasmalogen levels are strongly influenced by breastfeeding, providing a clear and actionable target for intervention^12^. Thus, the plasmalogen score was designed to emphasise a lipid pathway with both mechanistic relevance and translational potential, rather than a broader set of individual associations without a clear biological focus. Notably, infant plasmalogen score at 6 months of age was associated with BMI z-score at 4 years of age, independent of maternal BMI, suggesting that ether lipid status may have lasting metabolic implications.

These findings underscore the potential for early ether lipid exposure to shape long-term metabolic health. The high ether lipid content in human milk compared to formula may be one mechanism through which breastfeeding confers protection against obesity^13^. Breastfeeding promotion remains a critical public health strategy, especially for populations at higher risk of maternal obesity. At the same time, supplementation strategies (including maternal dietary supplementation during pregnancy or infant formula reformulation) could offer additional ways to optimise ether lipid availability in early life. Plasmalogen score and ether lipids in early life warrant further investigation as potential tools to reduce intergenerational obesity risk by targeting metabolic programming early.

### Strengths and limitations

The integration of a well-characterised birth cohort (BIS) with comprehensive lipidomics data and advanced statistical methods, including mediation analysis, is a major strength of this study. The mediation analysis included 551 mother-child dyads with complete datasets, larger than other studies to date, and 6 lipids were identified for mediating the relationship between maternal pp-BMI and infant birth weight. Use of pp-BMI and infant BMI are limited in their interpretation; however, they were the most appropriate growth measures in the context of lipidomics. Prior studies indicate that pp-BMI is a strong predictor of offspring obesity risk and that infants with high BMI in early life have higher obesity risk as they age^43–45^. While lipid metabolism was the primary focus of this study, we acknowledge that other biological mechanisms, including inflammation and hormonal pathways, may also contribute to intergenerational obesity risk^5^. While our analyses treat maternal BMI as the upstream determinant, it is important to note that lipid metabolism and adiposity are tightly interlinked and may influence one another over time. The relationship between maternal BMI and lipid composition is therefore likely bidirectional, meaning their effects on infant outcomes are inherently linked. Given the limited understanding of lipid metabolism and trajectories in early life, we focused our main analyses on cross-sectional models to maximise sample size and statistical robustness. This cross-sectional nature of the analyses, including maternal 28-week gestational lipids, limited causal inference. Furthermore, it is not possible to unravel all environmental factors that will also be at play in this study. It remains possible that plasmalogen levels influence growth, metabolism, or other aspects of development at later stages beyond those captured in this study. Our findings need to be developed and validated in additional longitudinal and more diverse cohorts, with later follow-up of participants to ensure that links hold.

## Conclusion

Maternal pre-pregnancy BMI significantly impacts gestational lipid profiles and has downstream effects on infant birth weight and breastfeeding, which are obesity risk factors. Plasmalogen scores appear to reflect a maternal metabolic state that supports protective lipid programming in the infant, offering promising targets for post-natal intervention. Strategies to optimise ether lipid levels, including maternal dietary supplementation, breastfeeding promotion, and early life infant dietary supplementation, could provide a practical, modifiable way to enhance metabolic resilience and reduce obesity risk, improving metabolic health outcomes for future generations.

## Supplementary Information

Below is the link to the electronic supplementary material.

Supplementary Material 1

Supplementary Material 2

## Data Availability

Access to the BIS data used in this paper may be requested through the BIS Steering Committee. Requests to access cohort data are considered on scientific and ethical grounds and, if approved, provided under collaborative research agreements.
