# Supplementary material for: Lipid pathways connecting maternal BMI with infant obesity risk
Source: Sci Rep. 2025 Dec 30;16:438. doi: 10.1038/s41598-025-30081-7 (PMC12775464; doi:10.1038/s41598-025-30081-7)
Supplement: Supplementary file 1 — Supplementary Material 1 [file 41598_2025_30081_MOESM1_ESM.docx]

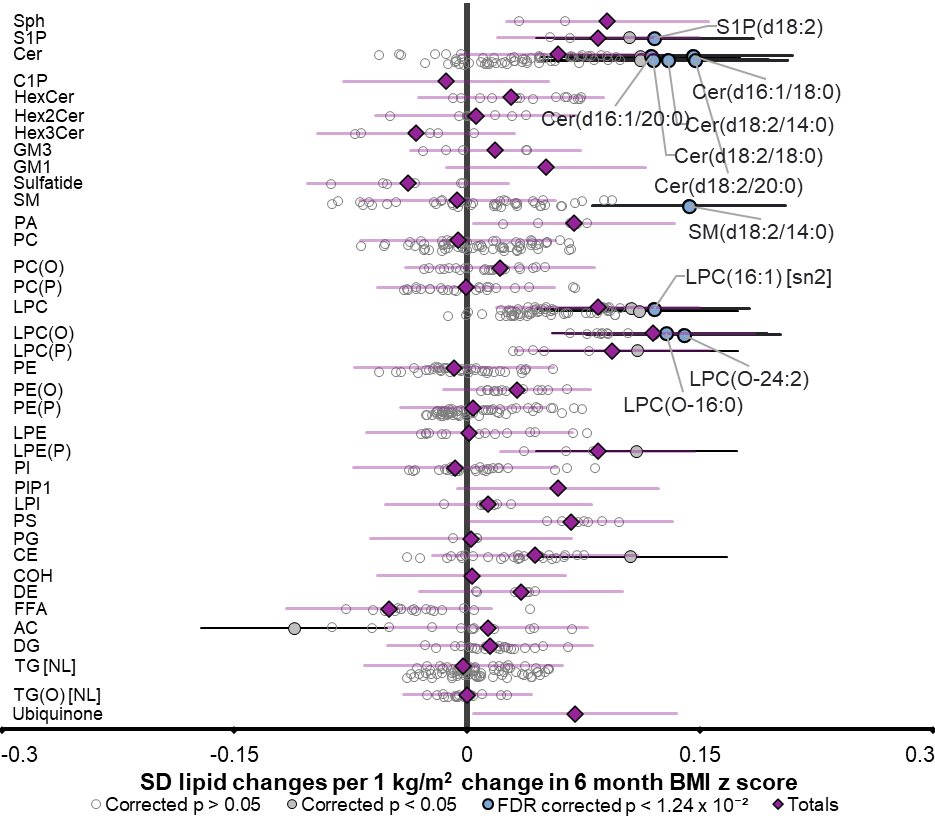


**Supplementary Figure 1. Infant 6 month lipidome associations with infant 6 month BMI z-score.** Linear regression analysis between 6-month lipidome and BMI z-score was performed adjusting for infant sex and breastfeeding status (n=723). Each circle represents an individual lipid species, open circles represent p > 0.05, white closed grey circles represent corrected p < 0.05, the top 10 most significantly associated lipid species are shown in blue and labelled. Purple diamonds represent lipid class totals. All p values were corrected for multiple comparisons (Benjamini and Hochberg correction). Horizontal bars indicate 95% confidence intervals for significant species.


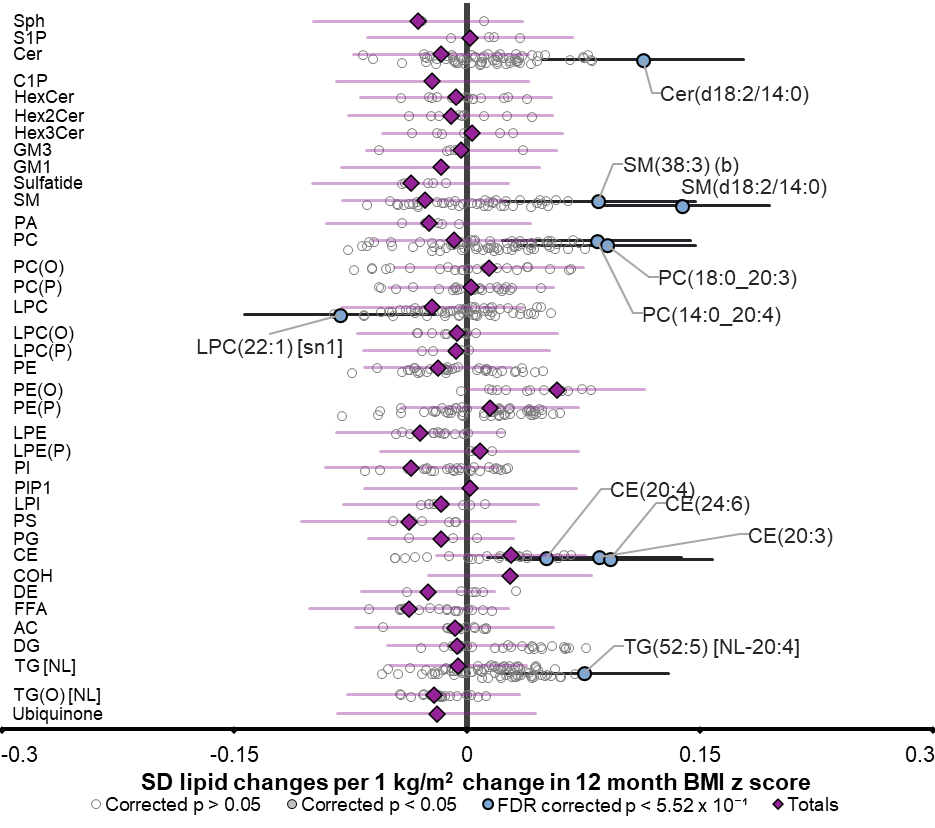


**Supplementary Figure 2. Infant 12 month lipidome associations with infant 12 month BMI z-score.** Linear regression analysis between 12-month lipidome and BMI z-score was performed adjusting for infant sex and breastfeeding status (n=704). Each circle represents an individual lipid species, open circles represent p > 0.05, white closed grey circles represent corrected p < 0.05, the top 10 most significantly associated lipid species are shown in blue and labelled. Purple diamonds represent lipid class totals. All p values were corrected for multiple comparisons (Benjamini and Hochberg correction). Horizontal bars indicate 95% confidence intervals for significant species.


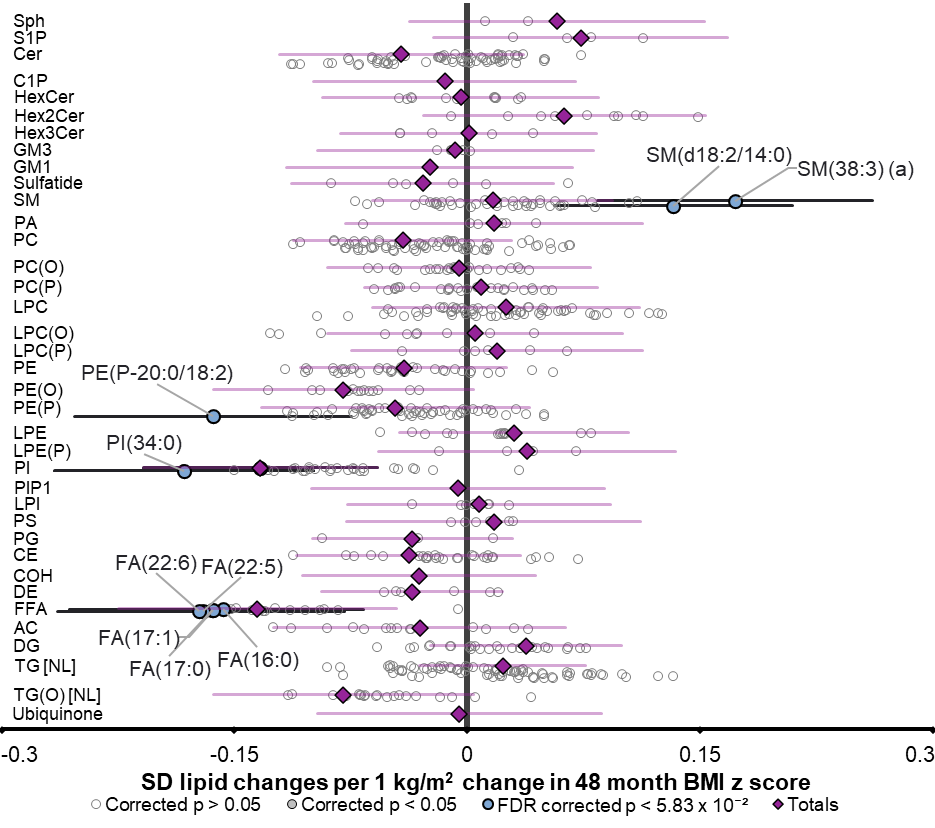


**Supplementary Figure 3. Infant 48 month lipidome associations with infant 48 month BMI z-score.** Linear regression analysis between 48-month lipidome and BMI z-score was performed adjusting for infant sex and breastfeeding duration (n=456). Each circle represents an individual lipid species, open circles represent p > 0.05, white closed grey circles represent corrected p < 0.05, the top 10 most significantly associated lipid species are shown in blue and labelled. Purple diamonds represent lipid class totals. All p values were corrected for multiple comparisons (Benjamini and Hochberg correction). Horizontal bars indicate 95% confidence intervals for significant species.
